# New Approaches for the Study of Orexin Function

**DOI:** 10.1111/j.1365-2826.2010.02015.x

**Published:** 2010-07

**Authors:** A Yamanaka, T Tsunematsu

**Affiliations:** * Section of Cell Signaling, Okazaki Institute for Integrative Bioscience, National Institute of Natural SciencesOkazaki, Japan; † National Institute for Physiological Sciences, National Institute of Natural SciencesOkazaki, Japan; ‡ Department of Physiological Sciences, The Graduate University for Advanced StudiesOkazaki, Japan

**Keywords:** neuropetides, receptors, membrane/nuclear, noradrenaline, 5-HT, CCK, vasopressin

## Abstract

Orexin is a neuropeptide produced by a specific subset of neurones located in the lateral hypothalamic area. Mice lacking either prepro-orexin or orexin receptor 2, as well as those in which orexin-producing neurones (orexin neurones) are deleted, share a common phenotype: altered sleep–wake regulation and the sudden onset of muscle atonia. These symptoms are similar to the human sleep disorder narcolepsy. In this review, we describe recent advances in the study of orexin function with a particular emphasis on microscopic techniques that better characterise the neuronal networks involving orexin neurones, as well as recent optogenetic approaches that allow for the activation or inhibition of specific neurones by expressing different light-activated proteins. In particular, the use of *orexin/halorhodopsin* and *orexin/channelrhodopsin-2* transgenic mice has demonstrated an important role for orexin neurones in regulating the sleep–wake cycle and state of arousal *in vivo*. Further refinement of these *in vitro* and *in vivo* techniques will allow for a more detailed understanding of the interaction of orexin with other neurotransmitter pathways in the brain.

Orexin-A and -B (also known as hypocretin 1 and hypocretin 2) are neuropeptides expressed exclusively by the neurones of the lateral hypothalamic area (LHA) (1, 2). Orexin-producing neurones (orexin neurones) project throughout the brain. Dense projections of orexin neurones are observed in the serotonergic dorsal raphe nucleus (DR), noradrenergic locus coeruleus (LC) and histaminergic tuberomammillary nucleus (TMN), and all of these nuclei are involved in promoting arousal. Although orexin mRNA is not expressed in the pituitary gland (3), orexin-positive nerve endings have been detected in the pituitary gland and paraventricular nucleus (4).

Orexin receptor-1 and -2 (OX1R and OX2R) are G-protein coupled receptors, and orexin binding is excitatory for postsynaptic cells (4–8). The effects of orexin on feeding behaviour were examined shortly after its discovery because the site of orexin neurones (i.e. the lateral hypothalamic area) is considered to be a feeding centre. Intracerebroventricular injection of orexin induced feeding behaviour, and food deprivation increased levels of prepro-orexin mRNA in rats (1, 9). Co-administration of the neuropeptide Y (NPY) Y1 receptor selective antagonist BIBO3304 significantly reduced orexin-induced feeding behaviour, indicating that orexin-induced increases in food intake were partially mediated through the NPY pathway (9). Thus, there are good data available indicating that orexin plays a role in regulating feeding behaviour; however, mice lacking the orexin peptide (*prepro-orexin* knockout mice) or OX2R (*OX2R* knockout mice), as well as those in which orexin-expressing neurones were deleted (*orexin/ataxin-3* transgenic mice), demonstrate a phenotype remarkably similar to the human sleep disorder narcolepsy (10–12). Additionally, *prepro-orexin* mRNA was not detected in the brain of a human with narcolepsy (13). Taken together, these data suggest that the orexin system plays a critical role regulating the sleep–wake cycle, and it appears to be particularly important for maintaining wakefulness.

In this review, we discuss several recent studies of transgenic mice in which orexin neurones express different fluorescent molecules, including enhanced green fluorescent protein (EGFP; *orexin/EGFP* mice), a calcium-sensing protein, yellow cameleon (*orexin/YC2.1* mice), and a retrograde tracer, tetanustoxin C-terminal fragment (*orexin/TTC::GFP* mice). Additionally, a novel optogenetic approach has demonstrated the importance of neuronal activity of orexin in the regulation of sleep/wakefulness *in vivo*.

## Regulatory mechanisms of orexin neurones revealed through the slice patch clamp technique

**Fig. 1:**
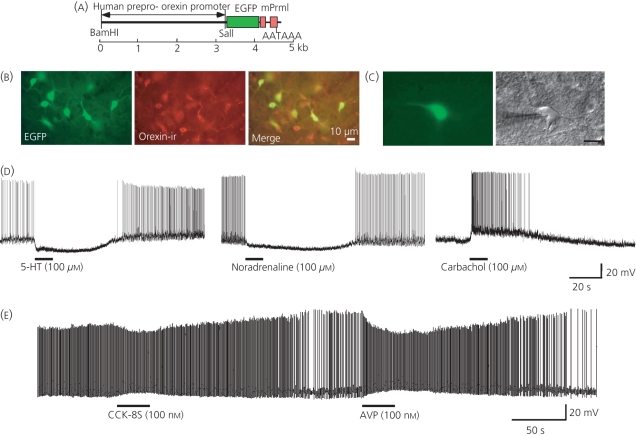
Electrophysiological analyses of orexin neurones using *orexin/EGFP* mice. (a) Structure of the *orexin/EGFP* transgene. (b) Orexin immunoreactive (-ir) (middle) and enhanced green fluorescent protein (EGFP)-positive (left) neurones are located in the lateral hypothalamic area, and colocalise in the merged image (right). (c) Identification of orexin neurones using EGFP fluorescence in a hypothalamic slice preparation from an *orexin/EGFP* transgenic mouse. EGFP fluorescence (left) and infrared differential interference contrast (right) images (Scale bar = 20 μm). (d) Under current clamp mode, 5-hydroxytryptamine (5-HT) (100 μm) and noradrenaline (100 μm) application induced hyperpolarisation in all orexin neurones, but carbachol (100 μm), an acetylcholine receptor agonist, induced depolarisation in 27% of orexin neurones. (e) Under current clamp mode, cholecystokinin (CCK-8S) (100 nm) and arginine-vasopressin (AVP) (100 nm) application induced depolarisation and an increased firing rate in the orexin neurones. (b, c, d) Reproduced with permission [14].

Electrophysiological techniques (e.g. the slice patch clamp) have proved invaluable for the study of neuronal membrane properties and input pathways. However, the small number of orexin neurones (approximately 4000 cells/mice brain) sparsely distributed through the LHA make them poor candidates for electrophysiological analyses. Additionally, they lack morphologic and electrophysiological features that allow them to be easily distinguished from other neurones. To better identify orexin neurones, transgenic mice expressing EGFP under the control of the orexin promoter were generated (14, 15). The orexin promoter is a 3.2-kb region of DNA upstream of the human *prepro-orexin* gene (Fig. 1a). This genomic fragment has two phylogenetically conserved regions located 287 bp and 2.5 kb upstream of the transcription initiation site of the human prepro-orexin gene (16), and this promoter enables the expression of genes specifically in orexin neurones. In the brains of these transgenic mice, EGFP was expressed by approximately 90% of orexin neurones (Fig. 1b), and no ectopic EGFP expression was observed throughout the brain. Once generated, we were able to perform electrophysiological studies of orexin neurones (Fig. 1c), and we first examined the effects of the fast neurotransmitters, glutamate and GABA, on orexin neurones. Ionotropic glutamate receptor agonists (AMPA and NMDA) depolarised orexin neurones, but GABA_A_ (muscimol) and GABA_B_ (baclofen) receptor agonists hyperpolarised orexin neurones (14, 17). Additionally, neurotransmitters implicated in sleep–wake regulation were applied to orexin neurones. Serotonergic (5-hydroxytryptamine; 5-HT) neurones in the DR, noradrenergic neurones in the LC and histaminergic neurones in the TMN play important roles in the regulation of sleep and wakefulness. These nuclei are densely innervated by orexin neurones and activated by orexin (4–8), and Table 1 summarises the compounds reported to affect the activity of orexin neurones. Both 5-HT and noradrenaline hyperpolarise all orexin neurones through the G-protein-coupled inwardly rectifying potassium channels downstream of 5-HT_1A_ receptors and α2A receptors, respectively (Fig. 1d) (18, 19). Interestingly, histamine appears to have no effect on orexin neurones but, in contrast, carbachol, a muscarinic agonist, variably affects orexin neurones; carbachol activates 27% of orexin neurones and inhibits 6% of orexin neurones (Fig. 1d) (14, 20). The cholinergic nuclei (laterodorsal tegmental nucleus and peduncle pontine nucleus) have been implicated in the regulation of rapid eye movement (REM) sleep and arousal (21). Alternatively, cholinergic neurones in the basal forebrain may maintain wakefulness (22). Carbachol-induced activation of orexin neurones is mediated through M_3_ receptors (23), although the inhibitory receptors remain unknown. However, M_2_ and/or M_4_ receptors are likely to be involved because they are coupled to inhibitory Gi/Go type G proteins, whereas M_1_, M_3_ and M_5_ receptors are coupled to Gq/G_11_ type G proteins signalling molecules (24).

**Table 1:** Substances which affect the activity of orexin neurones.

Substances	Receptor	Reference
Activation		
Glutamate	AMPA, NMDA	(12)
Acetylcholine	M_3_	(12, 17, 20)
Cholecystokinin	CCK_A_	(22)
Arginine-vasopressin	V1a	(22, 23)
Oxytocin	V1a	(22, 23)
Neurotensin	?	(22)
Ghrelin	GHSR	(13)
Thyrotropin-releasing hormone	TRH-R1	(35)
Glucagon-like peptide-1	GLP-1R	(36)
Corticotrophin-releasing factor	CRFR1	(37)
ATP	P2X	(38)
CO_2_	?	(39)
H^+^	?	(39)
Inhibition		
GABA	GABA_A_, GABA_B_	(12, 14)
5-Hydroxytryptamine	5-HT_1A_	(15)
Noradrenaline	α2A	(16)
Dopamine	α2A	(16)
Acetylcholine	?	(12)
Glucose	?	(13, 40, 41)
Leptin	OB-R	(13)
Neuropeptide Y	Y1	(42)
Adenosine	A_1_	(43)
Nociceptin	NOP	(44)
Met-enkephalin	μ	(45)

## Neuropeptidergic regulation of orexin neurones

To better characterise endogenous ligands affecting the activity of orexin neurones, transgenic mice were generated that specifically express the calcium-sensing protein, yellow cameleon 2.1 (YC2.1) (*orexin/YC2.1* transgenic mice) in orexin neurones (25). This system allowed us to monitor the activity of several orexin neurones simultaneously using fluorescence resonance energy transfer in slice preparations. Neuropeptides are considered to play important roles regulating feeding, drinking and sleep/wakefulness (26–29). The effects of neuropeptides were examined using calcium imaging of orexin neurones. Among the tested neuropeptides, a sulfated octapeptide form of cholecystokinin (CCK-8S), neurotensin (NT), oxytocin and arginine-vasopressin (AVP) activated orexin neurones, but nociceptin inhibited the activity of orexin neurones (25). CCK-8S- and AVP-induced activation of orexin neurones was also studied by slice patch clamp experiments (Fig. 1e). CCK-8S-induced depolarisation and inward currents were mediated by the CCK_A_ receptor, and the increased intracellular calcium concentration observed after CCK-8S stimulation was the result of calcium influx through the nonselective cation channel (NSCC). By contrast, AVP-induced depolarisation and inward currents were mediated by the V1a receptor, which is coupled to Gq/G_11_ type G-proteins (30). The subsequent activation of phospholipase Cβ increases the intracellular calcium concentration by stimulating both calcium influx through the NSCC and calcium release from intracellular stores.

**Fig. 2:**
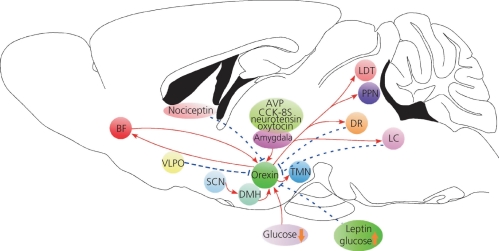
Interaction of orexin neurones with other neurones implicated in the regulation of sleep/wakefulness. The dashed blue line shows inhibitory projections, and the red arrow shows excitatory projections. AVP, arginine-vasopressin; BF, basal forebrain; CCK-8S, cholecystokinin; DMH, dorsomedial hypothalamic nucleus; DR, dorsal raphe nuclei; LC, locus coeruleus; LDT, laterodorsal tegmental nucleus; PPN, peduncle pontine nucleus; SCN, suprachiasmatic nucleus; TMN, tuberomammillary nucleus; VLPO, ventrolateral preoptic nucleus.

Behavioural experiments were performed to better understand the physiological significance of the AVP-induced activation of orexin neurones. Mice were generated in which Ataxin-3 (CAG repeat region in the ataxin-3 gene) was expressed under the control of the orexin promoter (*orexin/ataxin-3* mice), leading to the specific ablation of orexin neurones. Intracerebroventricular injection of AVP or water deprivation increased spontaneous locomotor activity in wild-type mice, but not in *orexin/ataxin-3* mice (10). AVP is an anti-diuretic hormone released during dehydration, and these results suggest that the orexin system plays a key role in water deprivation-induced hyperlocomotor activity. Additionally, a previous study showed that food deprivation induced hyperlocomotor activity, especially exploratory activity, through the activation of orexin neurones (15). Food deprivation is considered to decrease the extracellular glucose concentration, leading to increased ghrelin concentrations, and, ultimately, the depolarisation of orexin neurones (Table 1). By contrast, increasing extracellular glucose and leptin concentrations leads to hyperpolarisation of the orexin neurones (15) (Fig. 2). When considered together with the well documented role of orexin neurones in maintaining wakefulness, these results suggest that reduced food and/or water availability (in emergency situations) leads to increased arousal downstream of the activation of orexin neurones. Consistent with this, in mice placed on restricted feeding schedules (i.e. food access is limited to a few hours a day), orexin neurones become activated immediately preceding food access (31) and food-seeking behaviour is absent in orexin/ataxin-3 mice (32). However, the precise physiological role of orexin neurones in maintaining water or energy homeostasis remains unclear. It is possible that increased spontaneous locomotor activity and arousal may lead animals to expand their territories aiming to increase the chance of finding food or a new water source. It will be interesting to parse out the evolutionary implications of this system and its contribution to increased survival in nature.

By contrast, orexin neurones are activated by corticotrophin-releasing factor (CRF) (33) and thyrotropin-releasing hormone (TRH) (34) via the CRF R1 and TRH-R1 receptors, respectively. CRF-induced activation of orexin neurones might be involved in stress-induced increases in arousal or insomnia, whereas TRH-induced activation of orexin neurones may be related to locomotor activity, thermoregulation and sleep/wakefulness regulation.

## Afferent neurones to orexin neurones

To better understand the interaction of orexin neurones with other neurones implicated in the regulation of sleep/wakefulness, transgenic mice expressing a retrograde tracer, nontoxic C-terminal fragment of tetanus toxin (TTC::GFP), exclusively in orexin neurones were generated (20). TTC::GFP was selectively retrogradely transferred to interconnected neurones and transported to the cell bodies of higher-order neurones. Using this system, we were able to map upstream neuronal populations with direct synaptic connections to orexin neurones. TTC::GFP-labelled cells were identified in multiple areas of the brain, including serotonergic neurones in the median raphe nuclei and GABAergic neurones in the ventrolateral preoptic nucleus (VLPO), a known sleep centre. These observations suggest that neurones in these locations send inhibitory projections to orexin neurones because electrophysiological studies revealed that GABA and 5-HT inhibit all orexin neurones. Serotonergic innervation may act as a negative feedback system to maintain orexin neuronal activity within appropriate ranges. The sleep-active GABAergic neurones in the VLPO play an important role in the initiation and maintenance of slow wave sleep (SWS), and these neurones are activated by putative sleep promoting factors, including adenosine and prostagrandin D_2_ (35, 36). An electrophysiological study revealed that GABA inhibits orexin neurones through both GABA_A_ and GABA_B_ receptors (14, 17, 20, 37), suggesting that sleep-active neurones in the VLPO send GABAergic inhibitory projections to orexin neurones. This circuit may inhibit the activity of orexin neurones during sleep, and the reciprocal interactions between orexin neurones and these nuclei may be important for maintaining the balance of sleep and wakefulness.

In addition to known sleep centres, TTC::GFP labelled neurones were also found in the amygdala, infralimbic cortex, accumbens nucleus, lateral septum and bed nucleus of the stria terminalis. These regions contribute to the generation of mood and emotion, and neurones in these nuclei produce neuropeptides that activate orexin neurones such as CCK, NT and AVP. Inputs from these nuclei activate orexin neurones and may be involved in emotion-induced increases in arousal.

## Optical control of orexin neuronal activity *in vivo*

Taken together, slice patch clamp, calcium imaging and anatomical studies can be combined to generate a map of afferent neurones to orexin neurones (Fig. 2). Information obtained from these studies has expanded our understanding of the regulatory mechanisms governing sleep/wakefulness. However, it remains unclear how these networks interact *in vivo* to control the state of arousal of animals, and a recently developed optogenetics approach has already began to address these questions.

**Fig. 3:**
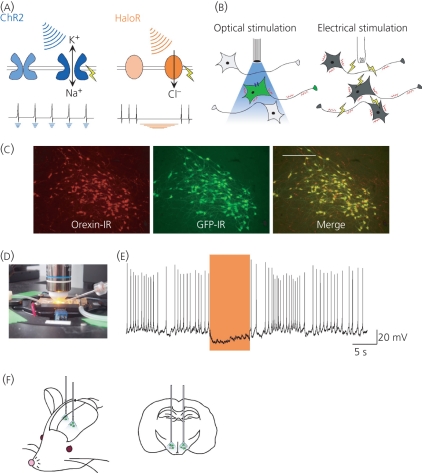
Control of orexin neuronal activity by light-activated proteins. (a) Activation of channelrhodopsin-2 (ChR2) by illumination with blue light induces depolarisation and generates action potentials (left). Activation of halorhodopsin (HaloR) by illumination with orange light induces hyperpolarisation and blocks action potentials (right). (b) Nonspecific stimulation by electrical stimulation (right). Specific neurones expressing light-activated proteins are stimulated by exposure to the appropriate wavelength of light (left). (c) HaloR is specifically expressed in the orexin neurones in the *orexin/Halo* transgenic mouse brain. Orexin immunoreactive (-ir) neurones are located in the lateral hypothalamic area (LHA) (red, right), and green fluorescent protein-positive neurones, indicating Halo expression, are also present in the LHA (green, middle). Scale bar = 500 μm. (d) Neurones are exposed to orange light through an objective lens. (e) Under current clamp mode, illumination with orange light induces hyperpolarisation and inhibits spontaneous action potentials in HaloR-expressing orexin neurones in the *orexin/Halo* transgenic mice. (f) To control the activity of orexin neurones, fibre optics (0.5 mm in diameter) are bilaterally implanted.

The microbial proteins channelrhodopsin-2 (ChR2) and halorhodopsin (HaloR) isolated from *Chlamydomonas reinhardtii* and *Natronomonas pharaonis*, respectively, respond to different wavelengths of light, and they have been used to control the polarisation of neurones after exposure to light (38–41). ChR2 is a monovalent cation channel, and exposure to blue light (∼470 nm) depolarises the membrane potential of ChR2-expressing cells. By contrast, HaloR is a chloride pump activated by orange light (∼580 nm). Activation of HaloR leads to an influx of chloride ions and hyperpolarises the membrane potential of HaloR-expressing cells (Fig. 3a). Thus, the expression of these proteins enables the control of neuronal activity by illuminating cells or animals with specific wavelengths of light, and these proteins can be expressed using tissue specific promoters in animals (Fig. 3b). Thus, ChR2 expression was genetically targeted to orexin neurones using lentiviral vectors in mice (42). In slice preparations, the firing frequency of ChR2-expressing orexin neurones was increased after exposure to pulsed blue light (up to 20 Hz). To study the role of these neurones *in vivo*, electroencephalographic (EEG) and electromyographic (EMG) electrodes were implanted in freely moving mice to determine the sleep/wakefulness stage, and fibre optics were implanted in the LHA to stimulate orexin neurones by light. Photostimulation of orexin neurones at a frequency of 5–30 Hz increased the total time of wakefulness and significantly reduced the amount of time spent in SWS and REM sleep in ChR2-expressing mice (42). This was the first study that used optogenetics to successfully control animal behaviour. These results suggest that orexin neurones can induce arousal in concert with other neurotransmitter systems such as the noradrenergic, serotonergic, cholinergic, histaminergic and dopaminergic systems. A subsequent study examined whether orexin-mediated sleep–wake transitions are affected by the light/dark period and sleep pressure. Photostimulation of ChR2-expressing orexin neurones was sufficient to increase the sleep–wake transitions for both SWS and REM sleep. Additionally, although photostimulation increased c-Fos expression in the orexin neurones consistent with an increase in neuronal activity, sleep deprivation (increasing sleep pressure) diminished the arousal effect induced by the photostimulation of orexin neurones (43). This study suggests that the activation of orexin neurones promotes wakefulness independently from light/dark period, but photostimulation of orexin neurones cannot overcome an increase in sleep pressure.

To further define the role of orexin neurones in regulating arousal, we examined whether silencing of the activity of orexin neurone affects sleep–wake transitions by generating transgenic mice in which orexin neurones express HaloR (*orexin/HaloR* mice). Immunohistochemical analysis confirmed that more than 90% of orexin-immunoreactive neurones express HaloR (Fig. 3c), and the functionality of this inactivation strategy was confirmed by slice patch clamp experiments performed *in vitro*. HaloR-expressing orexin neurones were identified using GFP, which was fused to the N-terminus of HaloR, and, under current clamp mode, the membrane potential of orexin neurones was recorded when orange light was applied through an objective lens (Fig. 3d). Exposure to orange light hyperpolarised the membrane potential by approximately 15 mV and blocked spontaneous firing by orexin neurones (Fig. 3e). Similar to the ChR2 experiments, fibre optics (0.5 mm diameter) were implanted bilaterally into the LHA, and EEG and EMG were simultaneously recorded to determine the sleep–wake state (Fig. 3f). Exposure of orexin neurones to orange light induced SWS in mice (i.e. decreased EMG and increased EEG delta power). Taken together, the ChR2 and HaloR experiments suggest that the stability of arousal may result from the activity of orexin neurones in conjunction with other arousal-promoting circuits, including the serotonergic DR, histaminergic TMN and noradrenergic LC.

Optogenetics is a powerful new tool allowing researchers to control the activity of specific neurones *in vitro* and *in vivo*. However, there are technical concerns that may limit the applicability of these techniques to all neurone types. In particular, *in vivo* photostimulation requires a strong light source such as a high power laser (100–1000 mW) and a fibre optics tethering system to guide the light to specific brain areas. However, such intense light may lead to thermal injury of local neurones. Optogenetic studies also require a specific and strong promoter to allow for the tight control of expression observed in orexin neurones. A combination of cre-loxP or FLP-FRT systems may enhance the expression of light-activated proteins under the control of weak promoters (44). Additionally, repeated and/or continuous exposure to light quickly inactivates the light-activated proteins, and this may be problematic for the application of optogenetics to relatively slow and long lasting physiological processes such as the control of neuroendocrine pathways. Although these problems need to be overcome for the broader applicability of these techniques, light-activated proteins are being rapidly discovered and engineered to increase their utility and potency (45, 46). Indeed, a new type of light-activated proton pumps, archaerhodopsin-3 (Arch), was very recently isolated from *Halorubrum sodomense* (47). Arch is a proton export pump and induces a more sustained and stable state of hyperpolarisation than HaloR. Optimisation of existing proteins and discovery of new molecules will improve optogenetical techniques and enable the more complete control of neuronal activity.

## Conclusions

Orexin neurones play important roles in the regulation of feeding, drinking, endocrine function and sleep/wakefulness. As discussed above, neural networks formed between orexin neurones and other neurones have been electrophysiologically and anatomically studied using several transgenic mice. Orexin neurones receive many excitatory and inhibitory inputs, and project to several sites after integrating these signals. However, it remains unclear how these networks interact and regulate animal behaviour *in vivo*. Our ability to control the activity of specific types of neurones *in vivo* using optogenetics will allow for the development of new research avenues to connect neural activity and behaviour.
